# The role of triterpenes in the management of diabetes mellitus and its complications

**DOI:** 10.1007/s11101-014-9369-x

**Published:** 2014-06-24

**Authors:** J. Nazaruk, M. Borzym-Kluczyk

**Affiliations:** 1 Department of Pharmacognosy, Medical University of Białystok, Mickiewicza 2a Str., 15-089 Białystok, Poland; 2 Department of Pharmaceutical Biochemistry, Medical University of Białystok, Mickiewicza 2a Str., 15-089 Białystok, Poland

**Keywords:** Diabetes mellitus, Triterpenes, Natural products

## Abstract

Diabetes mellitus is a chronic metabolic disease which is a serious global problem. In 2010 an estimated 285 million people had diabetes and within the next 20 years this value is expected to almost double. Many antidiabetic therapies focus on improving insulin sensitivity, increasing insulin production, and/or decreasing the level of blood glucose. Although a number of synthetic medicines are available, drugs of natural origin have aroused great interest. Triterpenes seem to demonstrate adequate properties. Many experiments have shown that these compounds have several antidiabetic mechanisms. They can inhibit enzymes involved in glucose metabolism, prevent the development of insulin resistance and normalize plasma glucose and insulin levels. These natural compounds, in contrast to synthetic drugs, apart from producing a hypoglycemic effect have also been found to manifest hypolipidemic and anti-obesity activity. Triterpenes are also promising agents in the prevention of diabetic complications. They have strong antioxidant activity and inhibit the formation of advanced glycation end products, implicated in the pathogenesis of diabetic nephropathy, embryopathy, neuropathy or impaired wound healing. Until now very few clinical studies have been concerned with the application of triterpenes in treating diabetes. However, due to their great therapeutic potential, these compounds deserve special attention.

## Introduction

**Fig. 1:**
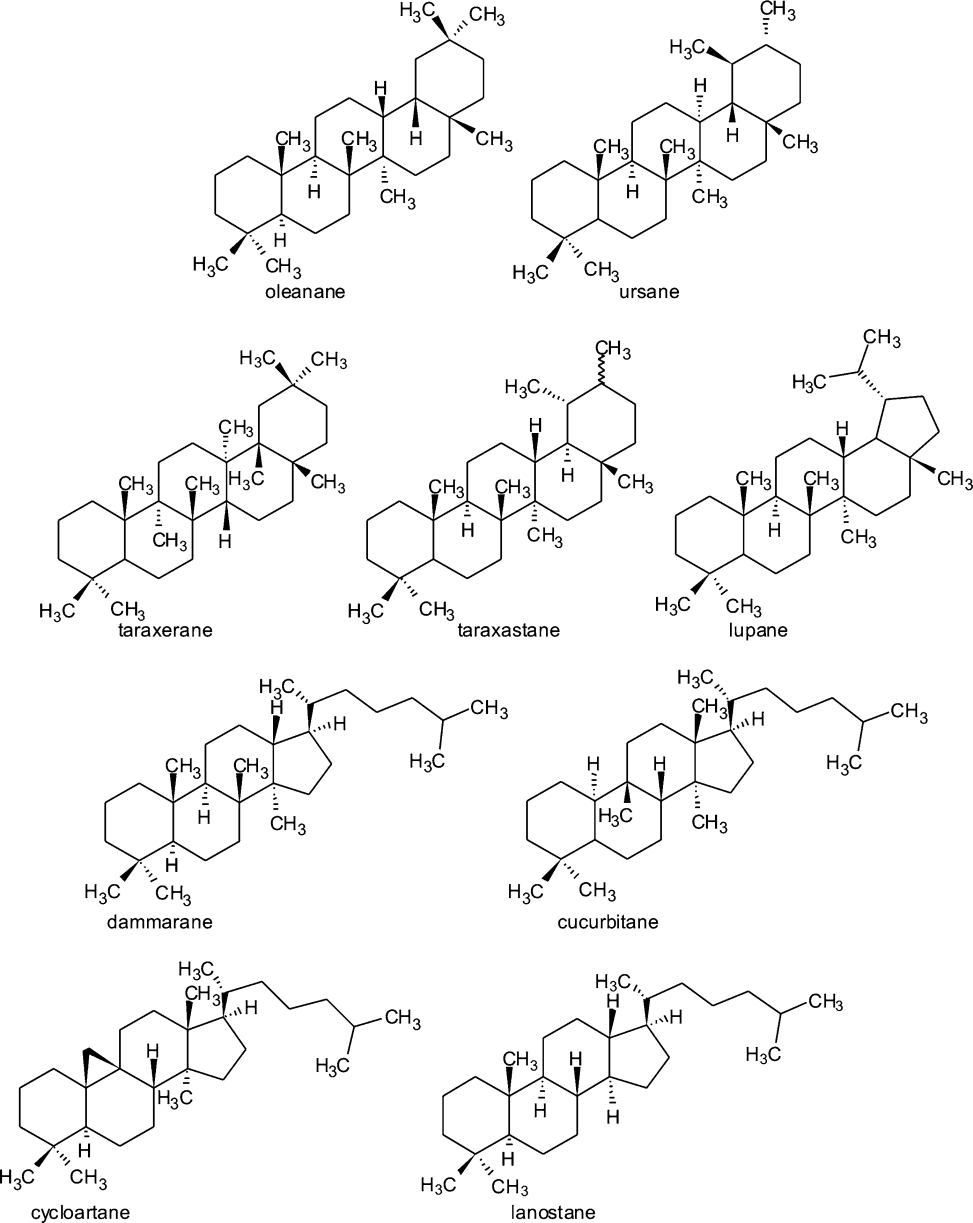
Chemical structures of the main subclasses of triterpenes

Triterpenes constitute a large structurally diverse group of natural compounds biogenetically derived from active isoprene. Two C_15_ units build squalene or related acyclic 30-carbon precursors. As the result of their cyclization and oxidation, various structures are formed. Transformations occur in two ways, one producing tetra- and pentacyclic triterpenes and the other one leading through cycloartenole to cucurbitacines or to cholesterol and farther to phytosterols, cardiac glycosides and steroid saponins. The most common structures of triterpenes include pentacyclic—oleanane, ursane, taraxerane, taraxastane, lupane, and tetracyclic—dammarane and cucurbitane (Fig. 1) (Sticher 2010). Another group consists of nortriterpenoids formed from tetracyclic triterpene precursors through oxidation and degradation, resulting in fewer than thirty carbon atoms in the basic skeleton. These are divided into two groups: limonoids (C_26_) and quassinoids (C_20_ and C_19_) (Harborne and Baxter 1993).

Triterpenes, especially pentacyclic ones, represent secondary metabolites that are widely distributed in the plant kingdom and found in leaves, stem bark, fruits and roots (Jäger et al. 2009). They are frequently the object of phytochemical and pharmacological investigations. The curative potential of triterpenes is very high yet still poorly recognized. Numerous in vitro and in vivo studies have revealed their multidirectional properties: anti-cancer (Laszczyk 2009), antioxidant (Ramachandran and Prasad 2008), anti-inflammatory (Yasukawa et al. 1996), anti-atherosclerotic (Sudhahar et al. 2007) or antiviral (Baltina et al. 2003).

Diabetes mellitus (DM) is a metabolic disease associated with disrupted insulin secretion or/and insulin action, resulting in high blood glucose levels. DM is primarily due to genetic or lifestyle factors, and creates a numerous therapeutic problems. Untreated diabetes can be the cause of many complications in retina, kidney or peripheral nerves and of macrovascular disturbances, such as ischemic heart disease and stroke (Ban and Twigg 2008). New drugs are still being sought to treat diabetic patients (Tahrani et al. 2011). Many natural triterpenoids seems to have promising antidiabetic properties. Their therapeutic possibilities and mechanisms of action are the subject of this review.

## Molecular target of triterpenes

### Triterpenes as α-glucosidase and α-amylase inhibitors

The therapeutic approach to treating type 2 DM is to decrease postprandial glucose levels. It can be achieved through the inhibition of *α*-glucosidases and *α*-amylases which delay the absorbance of carbohydrates in the intestine, leading to a decrease in the postprandial insulin level (de Sales et al. 2012). There are many in vitro investigations indicating the ability of various plant-derived triterpenes to inhibit *α*-glucosidase and *α*-amylase activity.

2,3-*seco*-20(29)-Lupene-2,3-dioic acid, obtained from leaves and twigs of *Fagus hayatae* (Fagaceae), showed inhibitory activity against *α*-glucosidase type IV (from *Bacillus stearothermophilus*), with IC_50_ equaling 62.1 μM (the positive control acarbose IC_50_ 23 nM) (Lai et al. 2012). Compounds isolated from the root bark of *Euclea undulate* (Ebenaceae), namely *α*-amyrin-3O-*β*-(5-hydroxy) ferulic acid—IC_50_ 7.76 μM correlating with those of the positive control, acarbose (IC_50_ 7.35 μM) and lupane—IC_50_ 14.69 μM, have been found to inhibit *α*-glucosidase type 1 from baker’s yeast (Deutschländera et al. 2011). Corosolic acid (1-hydroxyursolic acid) isolated from the leaves of *Lagerstroemia speciosa* (Lythraceae) shows bioactivity against *α*-glucosidase from yeast with IC_50_ 3.53 μg/mL, (acarbose IC_50_ 1.82 μg/mL). The remaining constituents of this plant—maslinic acid, oleanolic acid and 23-hydroxyursolic acid exhibit a lower activity where their IC_50_ equaled 5.52, 6.29 and 8.14 μg/mL, respectively. The research of kinetics showed that these triterpene acids inhibited the enzyme uncompetitively (Hou et al. 2009). Pistagremic acid, dammarane type triterpene obtained from galls of *Pistacia chinensis* var. *integerrima* (Anacardiaceae), shows potent enzyme inhibitory activity both against yeast (IC_50_ 89.12 μM, acarbose IC_50_ 780.21 μM) and rat intestinal (IC_50_ 62.47 μM, acarbose IC_50_ 38.92 μM) *α*-glucosidases (Uddin et al. 2012). The binding mode of pistagremic acid to the *α*-glucosidase was analyzed using molecular docking simulations. It has a proper molecular shape and size for forming hydrogen bonds with an important amino acid surrounding the catalytic site of this enzyme (Uddin et al. 2012). Several ursane and oleane type triterpenes isolated from the roots of *Sanguisorba tenuifolia* (Rosaceae) have been found to exhibit dose-dependent *α*-glucosidase inhibitory activity and their IC_50_ value ranged between 0.62 and 3.62 mM. The most active inhibitors of the enzyme were euscaphic acid and *p*-coumaroylursolic acid whose IC_50_ values were 0.67 and 0.62 mM, respectively, comparable with acarbose having an IC_50_ value of 0.79 mM (Kuang et al. 2011). Oleanolic and ursolic acids found in *Phyllanthus amarus* (Euphorbiaceae) inhibited porcine pancreatic *α*-amylase. The IC_50_ value for the mixture of these compounds in the 2:1 ratio was 4.41 μM, however the second compound was much more active than the first one (Ali et al. 2006). Oleanane-type triterpene bartogenic acid, isolated from seeds of *Barringtonia racemosa* (Lecythidaceae), demonstrated moderate inhibitory activity against both enzymes (*α*-amylase type VIB from porcine pancreas and *α*-glucosidase type I from baker’s yeast and intestinal enzyme). The IC_50_ value of bartogenic acid for the latter enzyme was 168.09 μg/mL (Gowri et al. 2007).

### The influence of triterpenes on aldose reductase

Under normoglycemia most cellular glucose is phosphorylated into glucose 6-phosphate by hexokinase. A minor part of nonphosphorylated glucose enters the alternate route of the glucose metabolism, the so-called polyol pathway. In the first step of this pathway aldose reductase catalyzes the reduction of glucose into sorbitol. In the second step sorbitol is subsequently converted to fructose by sorbitol dehydrogenase (Cheng and González 1986). Under normoglycemic conditions aldose reductase might function physiologically as a general housekeeping enzyme, but under hyperglycemia, when the metabolism of glucose through the polyol pathway is significantly increased, it leads to diabetic microvascular complications (Petrash 2004).

**Fig. 2:**
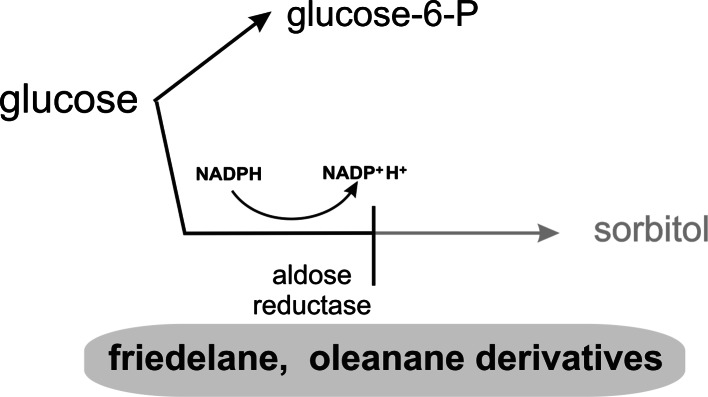
Inhibitory effect of triterpenes on sorbitol pathway

This process can be inhibited by some triterpene compounds (Fig. 2). Aldose reductase inhibitors were found among friedelane derivatives (kotalagenin 16-acetate) and isomeric oleanane derivatives (maytenfolic acid and 3*β*,22*α*-dihydroxyolean-12-en-29-oic acid), obtained from the roots of *Salacia oblonga* (Celastraceae). When they were used at a concentration of 100 μM the percentage of enzyme inhibition was 48.2, 54.6 and 75.9 %, respectively (Matsuda et al. 1999).

### Triterpenoids as protein tyrosine phosphatase 1B (PTP 1B) inhibitors

**Fig. 3:**
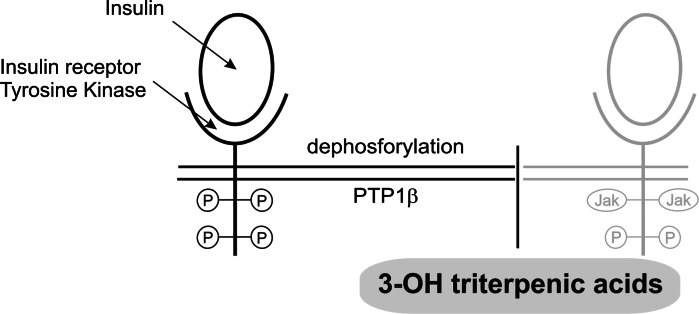
Triterpenes as PTP 1B inhibitors

Protein tyrosine phosphatases are enzymes which regulate cellular signaling and metabolism (Thareja et al. 2012). PTPases have been divided into two broad types, intracellular and transmembrane. PTP 1B belongs to a group of intracellular enzymes which cause negative regulation of insulin receptors as well as of the leptin signaling system. They are responsible for dephosphorylation process of the receptor *β*-subunit (Goldstein 2002). Inhibitors of PTP 1B can potentially ameliorate insulin resistance and normalize plasma glucose and insulin levels without inducing hypoglycemia (Thareja et al. 2012). Triterpenes with PTP 1B inhibitory activity are presented in Table 1 and their molecular target is demonstrated in Fig. 3.

**Table 1:** Triterpenoid inhibitors of PTP 1B

Compound	IC_50_ (μM)	Plant (family)	References
3-Oxoolean-12-en-27-oic acid	6.8	*Astilbe koreana* (Saxifragaceae)	Na et al. (2006a)
3*β*-Hydroxyolean-12-en-27-oic acid	5.2		
3*β*-Hydroxyurs-12-en-27-oic acid	4.9		
3*α*,24-Dihydroxyolean-12-en-27-oic acid	11.7		
3*β*,6*β*-Dihydroxyolean-12-en-27-oic acid	12.8		
Oleanolic acid	3.9	–	
	9.5	*Phoradendron reichenbachianum* (Viscaceae)	Ramírez-Espinosa et al. (2011)
	14.4	*Sambucus adnata* (Caprifoliaceae)	Sasaki et al. (2011)
3*β*-Hydroxyolean-12-en-28-oic acid	5.2	*Styrax japonica* (Styracaceae)	Kwon et al. (2008)
3*β*-Acetoxyolean-12-en-28-acid	7.8		
3*β*-Acetoxyolean-12-en-28-aldehyde	9.3		
Ursolic acid	2.3	*Phoradendron reichenbachianum* (Viscaceae)	Ramírez-Espinosa et al. (2011)
	3.08	*Cornus officinalis* (Cornaceae)	Zhang et al. (2006)
	3.8	*Symplocos paniculata* (Symplocaceae)	Na et al. (2006b)
	4.1	*Sambucus adnata* (Caprifoliaceae)	Sasaki et al. (2011)
	3.1	*Rhododendron brachycarpum* (Ericaceae)	Choi et al. (2012)
Moronic acid	13.2	*Phoradendron reichenbachianum* (Viscaceae)	Ramírez-Espinosa et al. (2011)
Morolic acid	9.1		
Corosolic acid	7.2	*Symplocos paniculata* (Symplocaceae)	Na et al. (2006b)
	7.0	*Rhododendron brachycarpum* (Ericaceae)	Choi et al. (2012)
2*α*,3*β*-Dihydroxy-24-nor-urs-4(23),11-dien-28,13*β*-olide (ilekudinol A)	29.1	*Weigela subsessilis* (Caprifoliaceae)	Na et al. (2010)
2*α*,3*β*-Dihydroxy-24-nor-urs-4(23),12-dien-28-oic acid (ilekudinol B)	5.3		
Rhododendric acid	6.3	*Rhododendron brachycarpum* (Ericaceae)	Choi et al. (2012)
(20*S*)-Dammarane-24(25)-ene-3*β*,20,21-tetrol	15.2	*Gynostemma pentaphyllum* (Cucurbitaceae)	Zhang et al. (2013)
(20*R*,23*R*)-3β,20-Dihydroxyldammarane-24-ene-21-oic acid-21,23-lactone	8.4		
3*β*-Hydroxyetio-17*β*-dammaranic acid	13.1		
Lupeol	5.6	*Sorbus commixta* (Rosaceae)	Na et al. (2009)
Lupenone	13.7		
Hopane-6α,22-diol	3.7	*Lecidella carpathica* (Lecanoraceae)	Seo et al. (2011)
Brialmontin 1	14.0

Some of the natural compounds examined were more active or had activity similar to RK-682 (IC_50_ 4.5 μM), which was used almost in all experiments as a positive control. Among the reviewed derivatives ursolic acid seemed to be the most active. This is important because it is a widely occuring pentacyclic triterpene (Sticher 2010).

The structure of the triterpenes plays a key role in inhibition of PTP 1B. As reported by Na et al. (2006a) and Kwon et al. (2008) the hydroxyl group at C-3 and the carboxyl group at C-28 or C-27 of the oleanane-type triterpenes are essential structural elements related to inhibitory activity. Similar to triterpenes isolated from leaves of *Rhododendron brachycarpum*, the hydroxyl group at C-3 in the ursane-type also seems indispensable for enzyme inhibition and the C-28 carboxyl group can form hydrogen bonds at the PTP 1B catalytic binding site (Choi et al. 2012). The inhibitory potency appears to become stronger when the lipophilicity of the tested compounds was increased (Choi et al. 2012). The main mode of action of triterpene acids isolated from leaves and stems of *Phoradendron reichenbachianum* was through PTP 1B enzymatic inhibition with the potent, reversible, selective and linear mixed-type inhibition models, and it is worth to noticing, that at a concentration of 50 μM enzyme activity was almost completely stopped (Ramírez-Espinosa et al. 2011). All compounds also showed moderate or weak activity toward other structurally related PTPases, such as the IF1, IF2 isoenzymes of human LMW–PTP, yeast LMW–PTP (LTP1) and human LAR (Ramírez-Espinosa et al. 2011). Ursolic acid, apart from PTP 1B, displayed obvious selectivity for other non-receptor-type PTPs–TCPTP (T cell protein tyrosine phosphatase) and SHP2 (src homology phosphatase-2), with IC_50_ levels of 3.33 and 2.73 μM, respectively (Zhang et al. 2006). TCPTP, SHP1 (src homology phosphatase-1) and SHP2 were also inhibited by corosolic acid, with their IC_50_ levels equaling 11.31, 24.56 and 10.50 μM, respectively (Shi et al. 2008).

Ilekudinol A and B isolated from *W. subsessilis* inhibited PTP1B in a non-competitive manner. This observation seems to indicate that a free carboxyl group at C-28 of 24-norursane triterpenes is essential to the inhibitory activity towards PTP 1B (Na et al. 2010). Lupane type triterpenes isolated from the stem bark of *Sorbus commixta* also inhibited PTP 1B in a non-competitive manner (Na et al. 2009).

### The influence of triterpenoids on glycolytic and related enzymes

Among glycolytic enzymes, glyceraldehyde-3-phosphate dehydrogenase (GAPDH), a key enzyme in glycolysis, plays a role in membrane fusion, phosphotransferase activity and apoptosis, whereas glycerol-3-phosphate dehydrogenase (G3PDH) catalyzes the reversible biological reduction in glycerone phosphate using NADH as a reducing equivalent to form glycerol-3-phosphate (Ishijima et al. 2008).

**Fig. 4:**
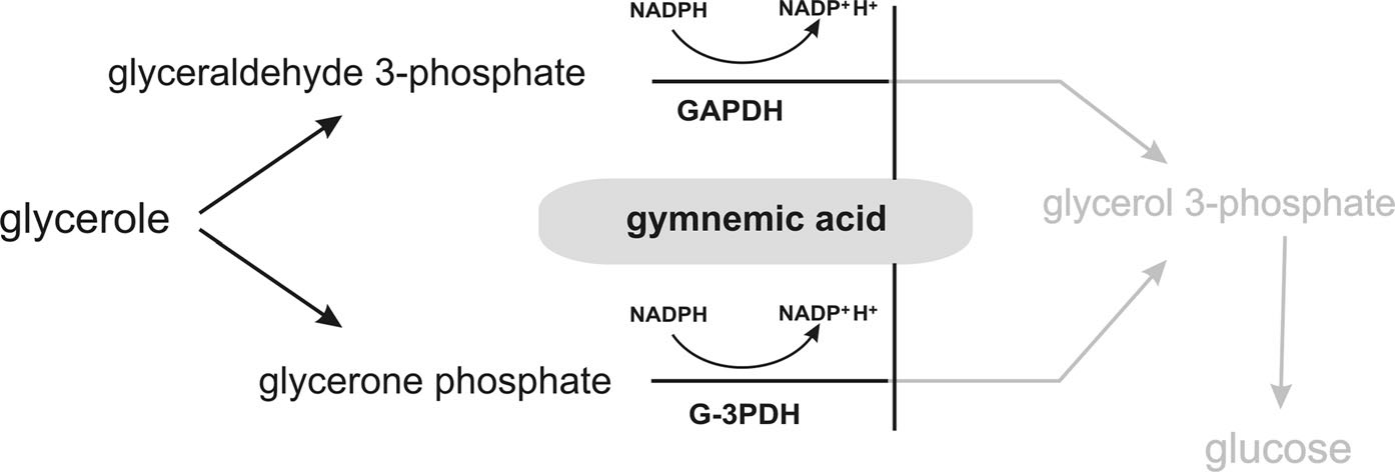
The influence of gymnemic acid on glycerol metabolism

The influence on these enzymes has been described for gymnemic acid, which is the mixture of approximately 10 oleanane-type tritepene saponins, found in leaves of *Gymnema sylvestre* (Asclepiadaceae). This mixture inhibited rabbit GAPDH and induced dephosphorylation of G3PDH and GAPDH (Fig. 4). It may have some physiological effects on glucose, glycerol and lipid metabolisms (Ishijima et al. 2008).

### Glycogen phosphorylase inhibitors

The level of glucose is regulated by hepatic glicogen phosphorylase (GP) catalyzing glycogenolysis resulting in an increased hepatic glucose output and glycogen synthase, which stimulates gluconeogenesis (Tahrani et al. 2011). GP exists in two interconvertible forms: dephosphorylated low-activity form, GPb, and phosphorylated high-activity form, GPa. In both forms, allosteric effectors can promote equilibrium between the less active T state and the more active R state (Oikonomakos et al. 2000). This enzyme is inhibited by insulin and activated by glucagon and other counter-regulatory hormones (Tahrani et al. 2011). It has been stated that in diabetes activity of GT is increased, therefore inhibitors of glycogen phosphorylase have been studied as potential therapy for attenuating hyperglycemia associated with type 2 diabetes (Baker et al. 2005).

**Fig. 5:**
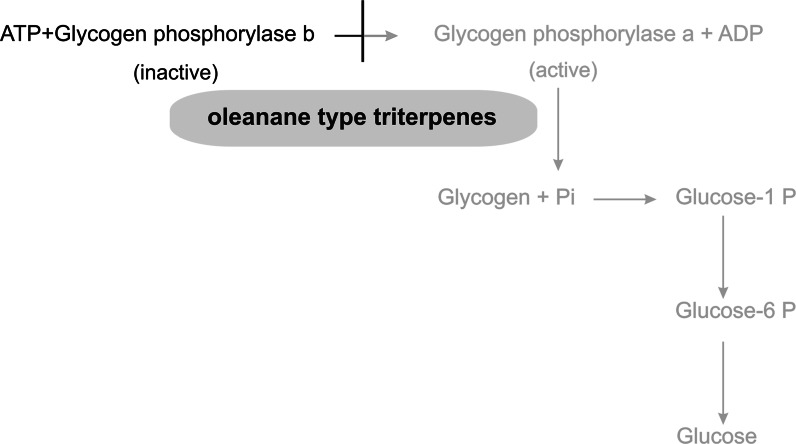
The action of triterpenes on glycogen phosphorylase

Among triterpenes most reports concern oleanane derivatives as potential GP inhibitors. Several such compounds were isolated from the roots of *Gypsophila oldhamiana* (Caryophyllaceae), with oleanolic acid and hederagenin being the most active GP inhibitors. At a concentration of 10 μM both compounds showed 73.07 % of GP inhibition, as compared to a similar compound, gypsogenin, with 45.11 % inhibition, whereas the positive control (caffeine in the concentration of 15 μM) showed 50 % inhibition of this enzyme (Luo et al. 2008). The authors, after comparing this with other compounds, suggest that the activity depends on the presence of a hydroxyl group at C-3 and CH_3_ or CH_2_OH groups at C-23 in the oleanane skeleton (Luo et al. 2008). Tormentic acid and asiatic acid obtained from the whole plant of *Potentilla biscolor* (Rosaceae) showed moderate (IC_50_ 90.5 μM, 65.4 μM, respectively), but higher than caffeine (IC_50_ 158 μM) inhibition of GP (Yang et al. 2010). On the other hand corosolic acid and maslinic acid were much more potent as liver GP inhibitors (IC_50_ 101 and 99 μM, respectively) than the positive control (caffeine, IC_50_ 648 μM) (Wen et al. 2005). The action of triterpenes on these enzymes is presented in Fig. 5.

### The influence of triterpenes on 11β-hydroxysteroid dehydrogenase type 1 (11β-HSD1)

11*β*-Hydroxysteroid dehydrogenase type I (11*β*-HSD1) is the enzyme that converts inactive 11-ketoglucocorticoids into active 11*β*-hydroxyforms in metabolically relevant tissues such as the liver, adipose tissue, skeletal muscles and pancreatic *β*-cells. Type II of this enzyme converts active cortisol into inactive cortisone, thereby preventing inappropriate mineralocorticoid receptor activation by glucocorticoids in aldosterone target tissues such as kidney, colon and salivary glands. Non-selective inhibition of 11*β*-HSD results in serious side-effects such as sodium retention, hypokalaemia and hypertension (Lipson et al. 2011). Chronically elevated local glucocorticoid action as a result of increased 11*β*-HSD1 activity is associated with metabolic syndrome, obesity, insulin resistance, type 2 diabetes mellitus and cardiovascular complications (Wamil and Seckl 2007). Selective inhibition of 11*β*-HSD1 has been proposed as a strategy to suppress glucocorticoid action in tissue specific manner. Many studies suggest that inhibition or down-regulation of 11*β*-HSD1 provides a decrease of excessive hepatic glucose production in hyperglycemia and diabetes mellitus, and exerts a positive effect on insulin sensitivity in diabetic subjects (Alberts et al. 2002; Stulnig and Waldhäusl 2004; Atanasov et al. 2006).

**Fig. 6:**
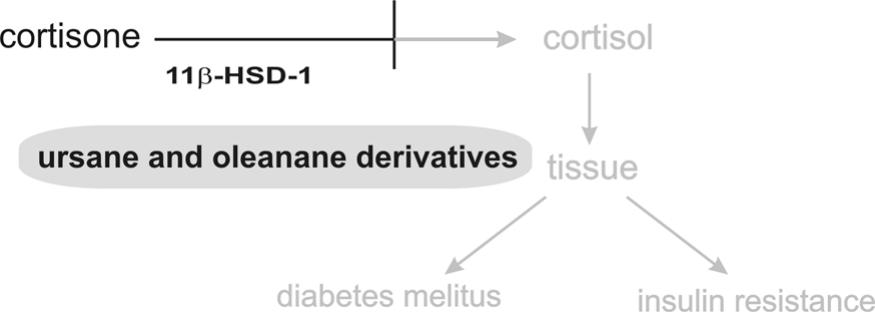
The role of triterpenes in cortisone metabolism

The ability to selectively inhibit11*β*-HSD1 (Fig. 6) has been demonstrated for many semisynthetic (nanomolar value of IC_50_) and natural ursane and oleanane derivatives (Blum et al. 2009). Strong activity has been exhibited by ursolic acid, 3-epicorosolic acid methyl ester, tormentic acid methyl ester and 2-*α*hydroxy-3-oxours-12-en-28-oic acid (IC_50_ 1.9, 5.2, 9.4 and 17 μM, respectively) isolated from leaves of *Eriobotrya japonica* (Rosaceae) (Rollinger et al. 2010). Interestingly the mixture of these compounds showed a significantly increased inhibitory potential on 11*β*-HSD1. An important role in ligand binding to the enzyme can be ascribed to carboxylic groups at C-17 and C-23, hydroxyl groups at C-2 and C-3, with the 2*S*,3*R* configuration seeming to be optimal (Rollinger et al. 2010). Constituents of *Bursera delpechiana* 11-keto-ursolic acid and 3-acetyl-11-keto-ursolic acid have also been reported as selective inhibitors of 11*β*-HSD1. Their IC_50_ values are equal to 2.06 and 1.35 μM, respectively (Rollinger et al. 2010).

### The influence of triterpenes on the TGR5 receptor and its implication in diabetes

TGR5, an emerging G protein-coupled receptor, was identified as a membrane receptor for bile acids. The expression of TGR5 and its function are distinct from the previously identified nuclear bile acid receptor, the farnesoid X receptor (FXR). These two bile acid receptors complement each other in maintaining bile acid homeostasis and mediating bile acid signaling. Both receptors also play roles in regulating inflammation and glucose metabolism (Chen et al. 2011). An interesting finding for TGR5 is its role in energy metabolism. The discovery of TGR5 expression in brown adipocyte tissues (BATs) and the recent discovery of BAT in the adult human body suggest a potential approach to combat obesity by targeting TGR5 to increase thermogenesis. The agonists of this receptor can also be used for the prevention of the development of insulin resistance in early stages of diabetes mellitus (Chen et al. 2011).

Oleanolic acid, a selective TGR5 agonist, which does not influence FXR is isolated from the leaves of *Olea europaea* (Oleaceae) (Sato et al. 2007). Other compounds with agonistic properties include betulinic acid and ursolic acid (Genet et al. 2010a). Betulinic acid is the most active (83 % efficacy with respect to litocholic acid used as positive control) of these three triterpenes mentioned above. The values of EC_50_ for oleanolic, betulinic and ursolic acids were 2.25; 1.04; 1.43 μM, respectively (Genet et al. 2010a). A comparison with other compounds having similar structure has proven that the hydroxyl group at C-3 plays a role in this type of activity. Unfortunately oleanolic acid has weak metabolic stability when administrated orally to rats and very low bioavailability (Sato et al. 2007; Genet et al. 2010a, b).

Nomilin, a highly oxygenated limonoid-type triterpene specific for *Citrus* sp., has also been recognized as an activator of TGR5. Its influence on TGR5 was higher than the natural agonist, chenodeoxycholic acid. Similar to the compounds mentioned above it does not induce FXR activity. Experimental animals treated with nomilin (0.2 %) had a lower body weight, decreased serum glucose and serum insulin, and an enhanced glucose tolerance (Ono et al. 2011).

## Tests on the antidiabetic activity of triterpenes

Experiments on the antidiabetic potential of natural products are carried out mainly in vivo, and are often complemented with in vitro studies to explore the mechanism of action of extracts or isolated compounds. Among animal models of DM those with pharmacologically induced diabetes and surgical or genetic models of diabetes are used. In vitro studies concern mainly insulin secretion and glucose uptake (Fröde and Medeiros 2008).

Many crude extracts of plants and isolated triterpenoids have been tested with regard to their antidiabetic and antihyperglycemic activity. Table 2 presents various cell models and Table 3 shows animal models adapted to test the antidiabetic activity of triterpenes.

**Table 2:** In vitro studies on antidiabetic activity of triterpenes

Plant (family)	Compound	Model	Result	References
*Momordica charantia* (Cucurbitaceae)	Karaviloside XI Momordicoside S 19-Epoxycucurbita-6-ene-23(*R*),24(*S*),25-triol 3-O-*β*-d-Glucopyranosyl 22(*S*),23(*R*),24(*R*), 25-tetrahydroxycucurbit-5-ene Momordicine II Kuguaglycoside G	L6 muscle cells, 3T3L1 adipocytes MIN6 *β*-cells	↑GLUT4 translocation ↑ AMPK ↑ Insulin secretion	Tan et al. (2008) Keller et al. (2011)
*Poria cocos* (Polyporaceae)	Pachymic acid	3T3-L1 adipocytes	↑ GLUT4 ↑ Phosphorylation of insulin receptor substrate (IRS)-1 ↑ Akt and AMPK	Huang et al. (2010)
*Panax ginseng* (Araliaceae)	Ginsenoside Rc	C2C12 myotubes	↑ Glucose uptake via activation of p38MAPK and AMPK thanks intracellular ROS generation	Lee et al. (2010)
*Campsis grandiflora* (Bignoniaceae)	Ursolic acid	CHO/IR cells 3T3-L1 adipocytes	↑ IR*β* auto-phosphorylation tyrosine ↑ Phosphorylation of the IR *β*-subunit, phosphorylation of Akt and glycogen synthase kinase-3*β* ↑ Insulin-stimulated GLUT4 translocation	Jung et al. (2007)
*Celastrus vulcanicola* (Celastraceae)	7*β*-Hydroxy-3-oxo-D:A-friedooleanan-28-oic acid (1) 7*β*,29-Dihydroxy-3-oxo-D:A-friedooleanane (2)	Huh7 cells (human hepatic cells)	↑ Phosphorylation of IR (in the absence of insulin ↑ Insulin-mediated IR tyrosine phosphorylation (only 1)	Ardiles et al. (2012)
–	Astragaloside IV	3T3-L1 adipocytes	↑ Insulin stimulated 2-DOG uptake, antagonized the TNF*α*-induced insulin resistance	Jiang et al. (2008)

**Table 3:** Antidiabetic activity of triterpenes tested in animal models

Plant (family)	Compound	Model	Result	References
*Momordica charantia* (Cucurbitaceae)	Momordicoside S momordicoside T	Insulin-sensitive and insulin-resistant mice	↑ Glucose tolerance ↑ Fatty acid oxidation	Tan et al. (2008)
*Eriobotrya japonica* (Rosaceae)	Extract containing above 50 % of triterpene acids (tormentic, corosolic, maslinic, oleanolic and ursolic acid)	Alloxan- and STZ-diabetic mice, HFD mice	↑ Serum insulin level ↑ SOD ↓ Glycosylated serum protein ↓ Total cholesterol and triglyceride reversing of insulin resistance	Lü et al. (2009), Shih et al. (2010)
*Poria cocos* (Polyporaceae)	Lanostane-type terpenoids: dehydrotumulosic acid, dehydrotrametenolic acid, pachymic acid	Diadetic db/db STZ-treated mice	↓ Postprandial blood glucose level ↑ Insulin sensitivity	Sato et al. (2002), Li et al. (2011)
*Protium heptaphyllum* (Burseraceae)	*α*- and *β*-amyrin	STZ-diabetic mice with HFD-induced hyperlipidemia	↓ Blood glucose, ↓ Total cholesterol and serum triglycerides	Santos et al. (2012)
–	Oleanolic acid	STZ-diabetic mice	↓ Glucose and triacylglycerides level ↓ Body weight ↓ Oxidative stress ↓ Gluconeogenesis in the liver mediated by the Akt/FoxO1 axis ↑ Insulin signal transduction in hepatocytes ↑ Glucose tolerance	Wang et al. (2011), Zeng et al. (2012)
–	Maslinic acid	KK-A^y^ mice (genetic type 2-diabetes)	↓ Blood glucose level ↓ Glycogenolysis via the inhibition of glycogen phosphorylase	Liu et al. (2007)
–	Asiatic acid	STZ-diabetic mice	↓ Blood glucose level ↓ Serum insulin level ↑ *β*-cell proliferation ↑ *β*-cell pro-survival signaling (protein kinase B/Akt kinase activation and Bcl-xL expression)	Liu et al. (2010)
–	Ursolic acid	STZ-diabetic mice	↓ Blood glucose level ↑ Plasma and pancreatic insulin concentrations preservation of pancreatic *β*-cells	Jang et al. (2009)
–	Astragaloside IV	HFD-STZ-diabetic mice	↓ Blood glucose level ↓ Blood insulin level ↓Hepatic GP and G6Pase activities	Lv et al. (2010)

The result of this review demonstrate that triterpenes often exert an effect on insulin biosynthesis, secretion and signaling. Additionally they also prevent pancreatic *β*-cell function, regulate total cholesterol and triglicerides level and body weight.

## Terpenoid sweetness inhibitors

It has been observed that subjects whose perception of sweetness had been decreased reduced consumption of total calories and sweet calories. This could be important in the prevention of uncontrolled glucose level elevation (Brala and Hagen 1983).

Sweetness inhibitors of terpenoid origin have been identified in some plants. These compounds were initially isolated from the leaves of *Gymnema sylvestre* and include oleanane-type saponins: gymnemic acid I–VI, X–XVIII and gymnema saponins. Unlike glycosides their aglycones are inactive. Such compounds, including ziziphin, jujubasaponins II–VI and dammarane type saponins are found in *Ziziphus jujuba* (Rhamnaceae). Dammarane derivatives, such as hodulcin, hodulosides I–IX and jujuboside B can be isolated from *Hovenia dulcis* (Rhamnaceae). Interestingly, the time for the recovery of the ability to taste sweetness differs for compounds from every plant. For *G*. *sylvestre* it is 15 min–24 h, for *Z. jujube* it is 5–10 min and for *H. dulcis* it is 1–4 min (Suttisri et al. 1995).

## Reduction of diabetes mellitus complications

### Antioxidants in diabetes mellitus

**Fig. 7:**
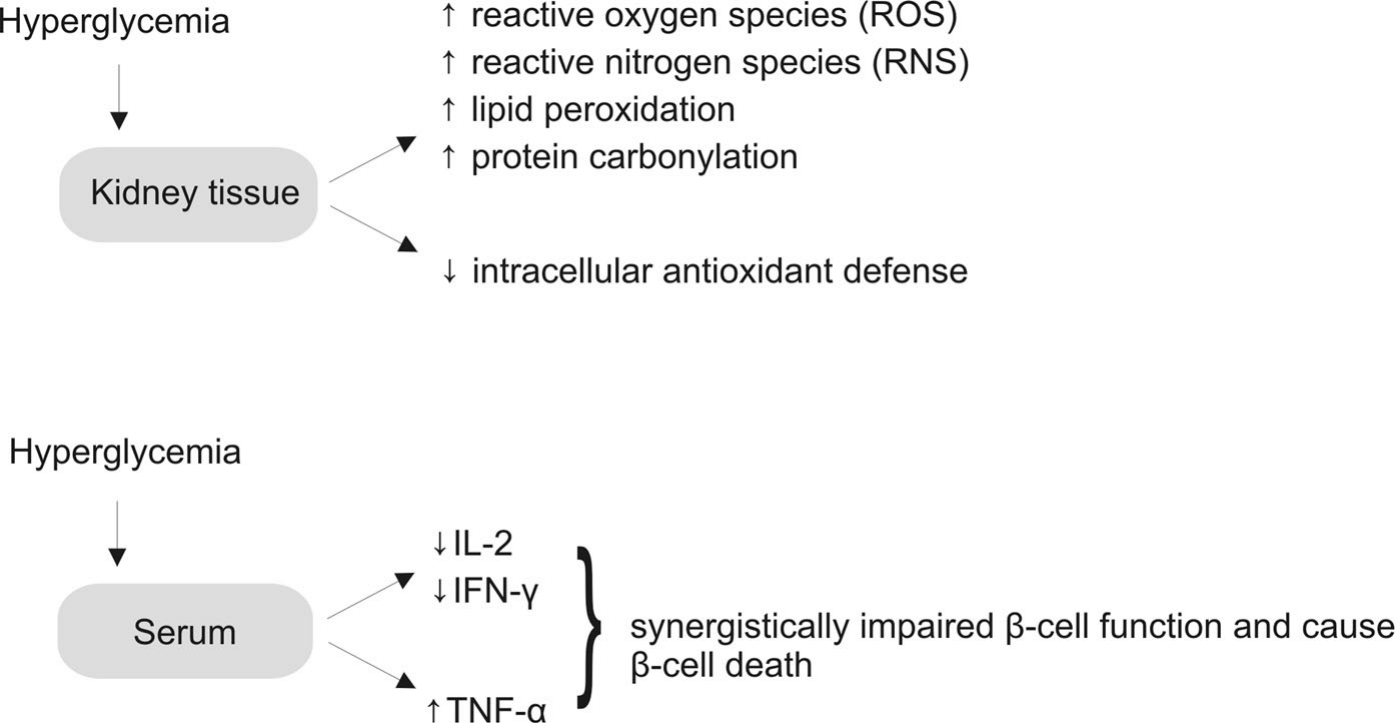
The scheme of consequences of hyperglycemia in kidney tissue and serum

It has been shown in various studies that diabetes mellitus is associated with the increased formation of free radicals and a decrease in antioxidant potential (Rahimi et al. 2005). Oxidative stress induced by hyperglycemia and free fatty acids causes insulin resistance, *β*-cell dysfunction, and late diabetic complications (Evans et al. 2002; Manna et al. 2009). Figure 7 shows disorders triggered by oxidative stress in kidney and serum.

The application of antioxidants especially of natural origin is one of the strategies in treating DM (Rahimi et al. 2005). Triterpenes play an important role as plant antioxidants. Ursolic acid exhibited hydroxyl radical scavenging activity, perhaps through its hydrogen donating ability. It also scavenged superoxide anions (Ramachandran and Prasad 2008). Corosolic acid reduced levels of thiobarbituric acid-reactive substance (TBARS) and 8-hydroxydeoxyguanosine (8-OHdG), both of which are oxidative stress biomarkers (Yamaguchi et al. 2006). Arjunoic acid present in the bark of *Terminalia arjuna* showed considerable activity against oxidative processes. This oleanane derivative prevented alternation of STZ-induced intracellular RNS and ROS formation in spleen tissue, deactivated the polyol pathway, enhanced the level of IL-2 and IFN-γ and decreased the level of TNF-α (Manna et al. 2010). The lupane-type triterpene, bacosine, obtained from the herb of *Bacopa monnieri* (Scrophulariaceae) also displayed antioxidant properties. This compound significantly decreased the level of malonylaldehyde, increased the level of glutathione (GSH) and the activity of superoxide dismutase (SOD) and catalase (CAT) in the liver of diabetic rats (Ghosh et al. 2011).

### The importance of triterpenes in AGE synthesis and in relieving complications provoked by them

In the etiology of diabetic vascular complications a major role is played by advanced glycation end products (AGEs) produced by non-enzymatic glycation and oxidation of proteins and lipids. AGEs have been implicated in the pathogenesis of diabetic nephropathy, embryopathy, neuropathy or impaired wound healing and independently may predict cardiovascular morbidity and stroke in the diabetic population (Ahmed 2005). Resulting AGEs activate the receptor for advanced glycation end products (RAGE). Activation of RAGE by AGEs causes upregulation of the transcription factor NF-*κ*B and its target genes. Binding of AGEs to RAGE induces a cascade of processes leading to damage of arteries in diabetic patients (Goldin et al. 2006). The role of RAGE has been observed in the development of accelerated atherosclerosis associated with diabetes (Basta et al. 2004).

Some triterpene compounds possess the ability to suppress formation of AGEs and are promising agents in the prevention and treatment of DM complications. Ursolic acid as a strong antioxidant suppressed oxidative stress and ameliorated vascular injury in STZ-induced diabetic rats through inhibition of activation of the RAGE-NADPH oxidase-NF-*κ*B signal transduction pathway (Xiang et al. 2012).

The effect of oleanolic acid and ursolic acid on AGEs production in renal tissue has been examined. Following treatment of STZ-diabetic mice with these compounds renal aldose reductase activity was suppressed and glyoxalase I activity was enhanced, which contributed to a decrease in renal AGEs formation and an improvement of renal functions (Wang et al. 2010). Oleanolic acid is a strong inhibitor of the formation of glycative products in vitro (Yin and Chan 2007). Also astragalosides and dammarane-type saponins from *Astragalus membranaceus* (Fabacea) were found to have an impact on the production of AGEs. An in vitro test showed astragaloside V to have the strongest inhibitory effect of several isolated compounds (Motomura et al. 2009).

### Other possibilities of triterpenes use

When HFD-diabetic mice were treated with ursolic acid at a concentration of 0.2 %, monocyte chemotactic activity was decreased in correlation with the extent of reduction in atherosclerotic lesion formation. This compound reduced monocyte transmigration and macrophage recruitment. The results suggest that ursolic acid protects monocytes from metabolic stress and inhibits the transformation of healthy monocytes into a hyper-chemotactic phenotype (Ullevig et al. 2011). Ursolic acid administrated to insulin-deficient diabetic mice at a dose of 0.01 %, as a food supplement prevented diabetic nephropathy through significant inhibition in 75 % of glomerular hypertrophy and suppression of type IV collagen accumulation in glomeruli. It also suppressed diabetes-induced activation of STAT-3, ERK1/2 and JNK pathways, and iNOS overexpression in kidney tissue (Zhou et al. 2010). A neuroprotective effect was observed in hyperglycemic rats treated with maslinic acid (5 or 50 mg/kg b.w.) after focal cerebral ischemia. A reduction in infarct volumes, improvement of neurological scores and enhancement of the glial glutamate transporter (GLT-1) expression at the protein and mRNA levels have been proven (Guan et al. 2011). A mixture of triterpene acids (with ursolic acid and oleanolic acid as main components) isolated from *Cornus officinalis* (Cornaceae) fruit suppressed upregulation of mRNA expression of the endothelin system, iNOS and other disorders of this type involved in the development of vascular abnormalities and retinopathy (Su et al. 2007).

## Bioavailability and toxicity of triterpenes

Triterpenes are large molecules and their penetration through cell membranes can be difficult. However, experiments revealed that they permeate into cells, even over the blood–brain barrier, and accumulat in large amounts in the liver. Moreover, chronic intake of triterpene-rich natural products increases their bioavailability and accumulation in circulation and tissues (Yin et al. 2012). It has been observed that the bioavailability of triterpenes can be improved by combining them with cyclodextrins (Cerga et al. 2011).

Natural medication mainly requires repeated intake of high doses of the substance. Therefore, toxicity of maslinic acid has been examined. The results obtained from acute and chronic intake of this compound indicate that it does not exhibit any adverse effects on the variables tested in mice (Sanchez-Gonzalez et al. 2013).

## Future of triterpenes as antidiabetic agents

A review of literature revealed a multidirectional effect of triterpenes. However, plants rich in these compounds are mainly applied in folk medicine, with an exception of the extract from the leaves of *Lagerstroemia speciosa*, containing corosolic acid as the main component. The extract is available as the preparation Glucosol™, standardized to contain 1 % corosolic acid (Judy et al. 2003).

Triterpenes can become prototypes for anti-diabetic drugs. Based on the triterpene skeleton derivatives which are more active than their substrates can be synthesized, e.g. oleanolic acid dihydroxy-olide is a stronger *α*-glucosidase inhibitor than the initial substance (Ali et al. 2002) and a derivative obtained in the reaction of maslinic acid with 1,4-dibromobutane is a more potent glycogen phosphorylase inhibitor than maslinic acid itself (Wen et al. 2006).

## Conclusion

The majority of the plants described above containing triterpene compounds are used in various countries in traditional medicine as antidiabetic remedies. Contemporary experiments confirm their activity and, in many cases, explain the mechanisms. Selected triterpenes could become important remedies for curing diabetes mellitus and are promising compounds for the development of new multitarget bioactive drugs. The use of triterpenes as AGEs inhibitors may be a potentially effective strategy to prevent diabetic complications. Their activity has been demonstrated in a number of in vitro studies and on animal models, but continuous clinical research does not exist.

## Abbreviations

AMPK: AMP-activated protein kinase
CHO/IR: Chinese-hamster ovary cells expressing human IR
2-DOG: 2-Deoxy-d-[1-^3^H] glucose
GLUT4: Glucose transporter
HFD: High fat diet
IR: Insulin receptor
STZ: Streptozotocin
